# Adaptive deep SVM for detecting early heart disease among cardiac patients

**DOI:** 10.1038/s41598-025-15938-1

**Published:** 2025-08-18

**Authors:** S. N. Netra, N. N. Srinidhi, E. Naresh

**Affiliations:** 1 Manipal Institute of Technology Bengaluru, Manipal Academy of Higher Education, Manipal, India; 2 Department of Computer Science & Engineering, B.M.S. College of Engineering, Bengaluru, India

**Keywords:** Heart disease detection, Atrial fibrillation rate identification, Adaptive deep support vector machine, Adaptive multiscale convolution capsule network, Enhanced arbitrary variable-based ship rescue optimization, Disease prevention, Health services, Machine learning, Predictive medicine

## Abstract

Heart attack is one of the most common heart diseases, which causes more deaths worldwide. Early detection and continuous monitoring are essential in reducing the death rate caused by heart diseases. Machine learning gives a promising solution for early and accurate heart disease detection by analyzing the data from healthcare devices. Although existing studies have employed various machine learning techniques to detect heart disease, most of the techniques still face challenges in handling large healthcare datasets that affect the prediction outcomes. To solve this issue, the research work focuses on developing a novel framework for detecting heart disease in its early stages by using machine learning techniques. In the initial phase, the significant data required for the validation is collected from benchmark resources, and it is subjected to the weighted optimal features selection phase. Here, from the input data, the features are selected optimally and their weights are tuned using Enhanced Arbitrary Variable-based Ship Rescue Optimization (EAVSRO). Further, the optimally selected weighted features are fed into the detection phase. In this phase, an Adaptive Deep Support Vector Machine (AD-SVM) is employed to detect heart diseases. Once heart disease is detected, the Atrial Fibrillation (AF) rate is determined using the Adaptive Multiscale Convolution Capsule Network (AMCCNet). Finally, the AF rate is obtained from the developed AMCCNet, and its parameters are tuned using the same EAVSRO. Later, various experiments are performed in the recommended heart disease detection model over existing models to verify its effectiveness. The accuracy of the designed framework is 96.07%, which is enhanced than the other existing frameworks like CNN-LSTM, DCNN, Adaboost and SVM, respectively. Thus, the results proved that the developed model can effectively detect heart disease at the early stages and identify the AF rate, providing timely treatments.

## Introduction

Heart disease diagnosis is often challenging due to the various factors that can affect the heart’s function and structure, which creates challenges for medical professionals in diagnosing the disease more quickly and accurately^1^. When the heart becomes incapable of pumping enough blood to fulfil the body’s needs, it leads to heart failure. Cardiovascular Diseases (CVDs) are increasing day by day. These diseases significantly impact public health, thus affecting millions of individuals worldwide^2^. The main causes of heart failure are classified into two categories: the first one is problems with the heart’s structure, such as those caused by a previous heart attack, and the second one is problems regarding the function of the heart, like high blood pressure^3^. Swelling in the ankles and legs, shortness of breath and fatigue are the symptoms of heart failure. Heart failure is treated with the help of surgical procedures, medication and lifestyle modifications^4^. The inclusion of digital technologies in diagnosing heart diseases is crucial to aid healthcare providers in providing faster and more accurate diagnoses regarding heart conditions^5^. Heart disease diagnoses are done by a combination of tests, review of the patient’s medical history and physical examinations^6^. When doctors identify the symptoms of heart disease, they assess the clinical history of patients, conduct chest X-rays and perform physical exams. However, in some cases, the clinical signs and symptoms do not align with the results obtained from the standard diagnostic tools, which can lead to challenges in confirming a diagnosis^7^.

AF is the most common form of cardiac arrhythmia, and the process of early detection and treatment in terms of AF is complicated. This condition affects millions of people around the world as it increases the risk of mortality, heart failure, and stroke^8^. AF is particularly common in elderly people and leads to strokes and other cardiovascular diseases^9^. Due to the increase in stress levels and the luxurious lifestyle, the occurrence of CVDs is increasing rapidly. AF is commonly found in individuals with heart conditions, and it can also develop in younger people who have no previous heart disease^10^. Moreover, AF results in severe health conditions like heart failure and thromboembolism. These complications raise the mortality risk. Lifestyle choices and genetic factors play a significant role in the cause of AF^11^. Factors like excessive alcohol consumption, smoking, physical inactivity, obesity and a high-fat diet are some of the causes that develop AF conditions. The risks associated with AF are cerebral infarction and stroke; these diseases severely affect the health and lifespan of patients^12^.

One of the advanced fields of Artificial Intelligence (AI) is machine learning, and it is used in the healthcare industry for disease detection^13^. By processing and analyzing large amounts of data, machine learning algorithms are useful in different stages of medical diagnostics. The ability to detect CVD in its early stages helps to improve the patient’s health and helps to reduce the associated healthcare costs. Using classification algorithms for disease diagnosis is an important aspect of medical research as they perform data analysis more efficiently and cost-effectively^14^. However, despite the potential of machine learning to predict medical conditions, its application in predicting CVD survival rates among hypertensive patients using routine healthcare data needs more improvement^15^. There are three primary approaches to detecting AF: algorithms that focus on atrial activity, ventricular activity, and hybrid models that integrate both types of data. These algorithms generally extract relevant features from the heart’s activity and then make decisions based on threshold values or use techniques like random forests for detecting AF^16^. However, traditional deep-learning models are not capable of filtering out noise in clinical data and generalizing across huge volumes of patient records^17^. So, a heart disease detection model with an AF rate estimation model is developed to prevent other cardiac diseases.

The developed model gives the following contributions:

- To design an intelligent and automated heart disease detection framework integrated with an AF rate identification mechanism for improving early diagnosis results and providing timely treatments. The incorporation of deep learning-based strategies in this framework is capable of learning from complex and non-linear medical data patterns. This model enhances diagnostic accuracy by minimizing human errors. Also, this approach is helpful in preventing heart attacks or heart failure by determining heart disease precisely.
- To introduce an EAVSRO algorithm for performing weighted optimal feature selection. This algorithm extracts the most informative features from the medical dataset and optimizes the relevant weights. This feature selection process increases the discriminative power of essential features while reducing noise and redundant data from the dataset. By multiplying the optimized weights and features, the dimensionality of the data is effectively minimized, which leads to faster model training by decreasing the computational burden.
- To employ an AD-SVM model for efficient heart disease detection. This AD-SVM model has the combined advantages of accurate feature extraction and robust classification strength. This architecture learns the complex nonlinear relationships within the medical data for detecting the presence of heart disease. Moreover, the fine-tuning of AD-SVM’s parameters helps to solve problems like overfitting and the curse of dimensionality on imbalanced data.
- To develop an AMCCNet for precise AF rate estimation. The utilization of multi-scale convolutional layers captures the spatial features across varying resolutions, which is highly beneficial in analyzing diverse patterns present in medical data. The incorporation of capsule layers in AMCCNet effectively preserves spatial hierarchies between features that solve the data loss problem. Furthermore, parameter optimization of AMCCNet using EAVSRO enhances its learning ability and reliability of AF rate estimation.

The manuscript is organized as follows: In Module II, the related works regarding various heart disease detection models are given. The structural demonstration of the implemented heart disease detection model and the proposed algorithm is explained in Module III. In Module IV, the weighted optimal feature selection procedure and the implemented heart disease detection using AD-SVM are explained. The AF rate determination process is described in Module V. Module VI and Module VII discuss the experimental results and the conclusion.

## Literature survey

### Related works

In 2022, Kumar et al.^18^ have suggested a hybrid model by combining the Context-Aware Heuristics (CAH) and a deep learning mechanism known as DeepAware. When analyzing user-operated ambulatory Electrocardiogram (ECG) signals, the performance of the developed approach was high. This model used the contextual data from the ambulatory ECG for precise AF detection. The results ensured that the DeepAware model has high generalization ability and could facilitate long-term ambulatory surveillance for early diagnosis of AF by reducing the effort of clinicians.

In 2023, Chen et al.^19^ have classified AF using a 12-lead ECG dataset to train a deep learning approach. Continuous telemetry data from 984 patients were collected, and validations of the model were executed. The performance of this model on clean data attained high specificity scores. Furthermore, the computational requirements of the implemented AF detection were more feasible than existing approaches.

In 2024, Moltó-Balado et al.^20^ have utilized five machine learning models to recognize cardiovascular events in AF patients. For the training process, half of the data was used by employing different approaches and the prediction errors were minimized with the help of optimization. AdaBoost was considered the best detection model after the validation process.

In 2023, Ma and Xia^21^ have suggested a new AF detection algorithm using a Graph Convolution Network (GCN). Initially, the authors converted heartbeats into heartbeat diagrams for creating graph structures. The morphological details of each heartbeat were considered as the node of the graph, and the edge of the heartbeat was related to the time interval between heartbeats. The morphological and rhythmic characteristics of the ECG signals were then efficiently recovered, modelled, and fused in a single network for AF detection.

In 2024, Ben-Moshe et al.^22^ have detected AF episodes using ECG recordings. This approach used both the morphology and rhythm data for AF estimation. Two datasets with different lead positions, ethnicity and geography were considered in this work for analyzing the generalization performance.

In 2024, Ramesh and Lakshmanna^3^ suggested an Optimal Scrutiny Boosted Graph Convolutional Long Short-Term Memory (O-SBGC-LSTM) for detecting heart diseases. The suggested technique’s temporal receptive fields were expanded to reduce computation costs and improve the capacity to learn high-level semantic representations.

In 2023, Sarkar et al.^23^ have used a Convolutional Neural Network (CNN) for AF detection and ensemble features were combined to form an input signal, which was utilized by the CNN mechanism. The AF episode detection outcomes based on the features were highly accurate. Moreover, the applicability of the investigated model was tested on individual patient test datasets.

In 2023, Hossain et al.^24^ have implemented a hybrid deep learning mechanism for predicting heart diseases. The sequential learning was handled by the LSTM model, while the feature retrieval process was managed by the CNN model. As per the results, this combined method was highly useful in the early diagnosis process.

In addition to ECG-based and imaging-based approaches, Güven et al.^4^ proposed a mobile application framework for real-time heart disease diagnosis, emphasising portability and accessibility. Another work by Guven and Uysal^25^ explored long short-term feature extraction from heart sound data, providing a novel angle on cardiac signal processing using deep learning. Even though several techniques like Traditional Machine Learning (TML) algorithms and Ensemble Machine Learning (EML)^13^, machine learning-based framework^26^, and Least-Squares Support Vector Machine (LS-SVM)^27^ are developed to detect AF and heart diseases, they cannot capture the complex inter-feature dependencies present in the medical data. Traditional deep learning models like CNN or DNN mostly emphasize local patterns but cannot preserve spatial or hierarchical relationships between features that affect the robustness of AF rate determination. To solve these issues, this research work developed an innovative framework using machine learning and deep learning models. This approach can effectively detect heart disease and help with the precise and timely diagnosis of cardiac problems.

#### Problem statement

The symptoms of CVD are heart failure, heart attacks, blood artery blockage and various heart diseases that lead to death or other serious problems. Because of the degree of occultation associated with AF, many individuals do not have symptoms in the early stages. Diagnosis of heart disease is difficult due to the number of risk factors such as irregular pulse rate, excessive cholesterol, high blood pressure and hypertension. Early detection of CVD is crucial in lowering the mortality rate, and researchers have introduced many techniques for heart disease detection. Some of the limitations of existing heart disease detection models are provided below:

- Several existing heart disease prediction systems rely heavily on raw clinical datasets, and some of the models do not give importance to eliminating irrelevant or incomplete data. Thus, the precision of the heart disease detection process is affected and results in unreliable diagnostic outcomes.
- While some previous approaches have adopted basic feature selection methods, most of them fail to optimize the weights of selected features effectively. Due to the absence of weight optimization, the need for computational resources increases. Therefore, applying an efficient metaheuristic strategy for weighted feature selection becomes essential to ensure efficient processing.
- Conventional classifiers like decision trees rely on static decision boundaries, and they are not able to classify the complex variations present in clinical data. Similarly, traditional deep learning models often struggle to capture multi-scale spatial dependencies needed for AF rate identification. These conventional models often apply a single-layer classification boundary, so they fail to differentiate patients with minor and major symptoms.
- In many of the existing heart disease detection frameworks, once a disease is identified, the subsequent assessment of critical cardiac conditions such as AF is either ignored or performed using basic classifiers. Mostly, those approaches are not capable of retrieving temporal and spatial features. This results in an incomplete cardiac health evaluation of patients.

Positive and negative aspects of the existing models are given in Table 1.

**Table 1:** Positive and negative aspects of the existing models.

Author [citation]	Methodology	Features	Challenges
Kumar et al.^18^	CNN-LSTM	• It retrieves contextual features for precise atrial activity analysis. • Network complexity is very low due to the shared weight process.	• The time needed for analyzing ECG data is high, and it is computationally expensive.
Chen et al.^19^	DCNN	• It handles larger sample sizes by preventing class imbalance issues.	• Performance degradation issues occur because it is not capable of reducing noise in data.
Moltó-Balado et al.^20^	Adaboost	• It solves overfitting issues and protects the privacy of patients’ data.	• It could not handle multimodal images.
Ma and Xia^21^	GCN	• The rhythm features and morphological features are retrieved for detecting the AF condition.	• The time taken for the detection process is high.
Ben-Moshe et al.^22^	RawECGNet	• It removes the noisy windows during the pre-processing for obtaining accurate AF episode detection outcomes.	• Accuracy in the result changes based on the quality of ECG recordings.
Ramesh and Lakshmanna^3^	O-SBGC-LSTM	• It efficiently learns high-level semantic representation due to the expanded temporal receptive fields. • Computation cost is low.	• Interpretability issues arise while analyzing complex patterns.
Sarkar et al.^23^	CNN	• It can handle two-dimensional input images without computation issues.	• The risk of overfitting is low. • The need for the number of trainable parameters is high.
Hossain et al.^24^	CNN-LSTM	• It is capable of offering personalized treatment plans by identifying the factors influencing the development of CVD.	• Not applicable for detecting heart disease on larger datasets.

## An efficient heart disease detection framework with atrial fibrillation identification model using adaptive deep model

### Structural demonstration of implemented heart disease detection model

**Fig. 1:**
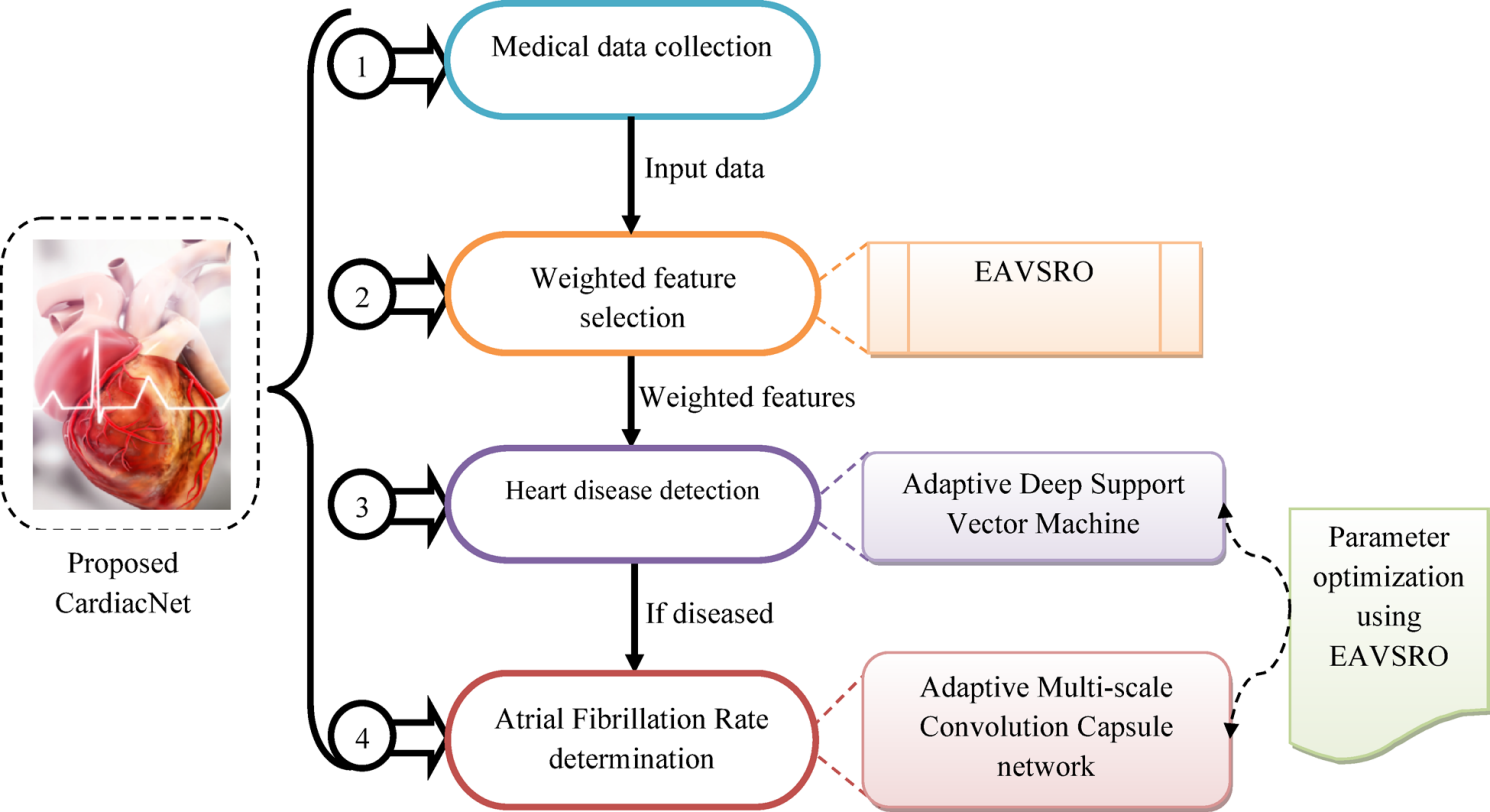
Structural Demonstration of the Proposed Heart Disease Detection Model.

The existing models, like DNN, are inefficient in handling structured medical datasets. Moreover, DNN models require a large amount of data for effective learning due to their complex architectures with many layers, and the chances of more computational burden and training time are high. Techniques like LSTM and CNN are less appropriate for non-sequential and static clinical medical data, which results in poor feature extraction. Furthermore, the prediction performance of models such as Adaboost is low, as this technique is not able to handle noisy, redundant, or irrelevant healthcare datasets. The absence of efficient feature selection and weighting techniques is a major drawback of these models that affects the accuracy of heart disease identification. To minimize complexity and increase the diagnosis accuracy of heart and atrial fibrillation diseases, a unique framework is developed. A structural demonstration of the proposed heart disease detection model is showcased in Fig. 1.

An intelligent framework using machine learning and deep learning models is developed for precise and timely diagnosis of cardiac problems. To lower the death rates and prevent other heart-related diseases, early diagnosis of heart conditions and AF is essential. Reducing the risk of stroke, heart failure, and other fatal conditions is another benefit of accurately diagnosing AF. Additionally, early diagnosis helps the medical staff to provide individualized patient care to improve the overall health outcomes of each patient. The input data is gathered from benchmark datasets for performing heart disease detection. Then, the features from collected data are retrieved using the EAVSRO algorithm to remove the irrelevant or noisy data. Using the same EAVSRO algorithm, the weights of the features are optimally determined, which helps to decrease the dimensionality of features and increase the processing efficiency. The optimal weights are multiplied by the retrieved features to obtain the weighted optimal features needed for heart disease detection. These features are passed to the proposed AD-SVM model for recognizing heart disease. This technique is beneficial in handling intricate, non-linear patterns in the weighted features. Accurate detection outcomes are attained by the parameter tuning process of EAVSRO in the AD-SVM model. The classification ability of SVM, along with the hierarchical feature learning efficiency of deep learning, is useful in analyzing the high-level features that showcase the presence of heart diseases. If a patient has heart disease, then the AF rate is determined using the implemented AMCCNet model. The cardiac risks of patients are efficiently identified by analyzing the AF rate of patients. The accuracy of AF rate prediction is increased by using the AMCCNet, as it is capable of extracting complicated hierarchical patterns and multi-level spatial features from medical data. The important patterns of the features are efficiently preserved by the AMCCNet while determining the AF rate. Using the EAVSRO, the parameters of AMCCNet are optimized to enhance the reliability of AF prediction. The suggested framework is evaluated using different performance metrics over conventional models to validate its ability in disease detection.

### Heart disease dataset representation

Two different datasets are considered in this research for the diagnosis of heart disease.

#### Dataset 1 (Heart Disease Dataset)

This dataset is accessed on 2025-04-08 from https://www.kaggle.com/datasets/johnsmith88/heart-disease-dataset. This dataset comprises 1026 instances, with 76 features, including 14 subsets of detailed information, such as blood pressure, sugar, cholesterol, and other relevant attributes. The class distribution of dataset 1 is imbalanced, with 500 instances belonging to the absence of the heart disease class and 526 instances belonging to the presence of the heart disease class. The file type of this dataset is .csv. The data is split into a training set of 821 (80% of the total) and a testing set of 205 data points (20% of the total).

#### Dataset 2 (heart disease)

This multivariate database is available on https://archive.ics.uci.edu/dataset/45/heart+disease, and it was accessed on 2025-04-08. This dataset contains 920 instances, with 76 features, including numerous attributes such as patient smoking history, chest pain details, and other relevant information. The class is distributed into five classes, namely normal, class 1, class 2, class 3, and class 4. Further, it has 4 databases, namely Cleveland, Hungarian, Switzerland, and VA Long Beach. Here, Cleveland contains 303, Hungarian has 294, Switzerland contains 123, and VA Long Beach has 200. The data is split into a training set of 736 (80% of the total) and a testing set of 184 data points (20% of the total).

It consists of both processed as well as unprocessed data files. The accumulated data for identifying heart disease is represented as${A_H}$and total data is represented by the variable $H$.

#### Ethical data usage notes

Ethical data usage in data collection prioritizes transparency, consent, and data minimization while ensuring fairness, accountability, and privacy protection. The ethical concerns addressed by the developed model are given below.

#### Data privacy

Above mentioned datasets are publicly available; each patient’s data is anonymized, and it has been complying with medical data protection regulations.

#### Consent

The proper patient consent was obtained during the data collection, rights and restrictions were checked, and compliance with data use agreements was ensured.

#### Bias considerations

The collected datasets are diverse, and representation, potential demographic biases and geographic and ethnic representation.

#### Security

The collected data are more secure.

### Presented EAVSRO

To choose the most relevant features from the dataset, the optimization process is done using the EAVSRO algorithm. This algorithm is designed by leveraging the strength of the traditional Ship Rescue Optimization (SRO) algorithm.

#### Reasons for choosing the SRO

SRO models the movement of ships during rescue operations, which allows it to explore the feature space in a structured and efficient manner. This helps in identifying relevant features while avoiding getting stuck in local optima, a common issue in many optimization algorithms. Further, it can handle large datasets with many features, which is crucial for many real-world applications.

The SRO^28^ algorithm mimics the ship rescue process. The large area search and focused search procedures help to avoid getting stuck in local optima.

1 $$x_{{j,i}}^{{new,q1}}=\left\{ {\frac{{{x_{j,i}}+r({q_{j,i}} - I{x_{j,i}}),}}{{{x_{j,i}}+r({x_{j,i}} - {q_{j,i}})}}} \right.\frac{{F{p_j}<{F_j}}}{{F{p_j} \geqslant {F_j}}}$$

Here, ${q_j}$is the position of prey for the $jth$ ship rescue, $F{p_j}$ is its objective function value, and $r$is a random number in the interval ${\text{[0, 1]}}$. But if the search space is highly complex and the solution spaces are poorly defined, then the performance of this algorithm will be affected.

#### Novelty

To solve this issue, a random number $r$is updated in the traditional SRO by using Eq. (2).

2 $$v=\frac{{RWS}}{{Csf+RWS+Bsa}}$$

Here, the variables$Bsa$, $RWS$and$Csf$denote the best fitness, worst fitness and best fitness values. These fitness functions help to explore the search space more effectively. The random number modification by considering the mean fitness value helps to find new regions in the search space. The chance of getting trapped in local optima is highly reduced by the new computation of random attributes. Better optimal solutions are achieved by the consideration of both worst and best fitness calculations for upgrading the value of random parameters. Finally, overfitting issues during AF rate prediction and heart disease detection can also be prevented by optimizing the model’s parameters with the suggested EAVSRO algorithm by using this updated random number, ensuring that the optimization process explores different regions of the parameter space, potentially leading to a better solution and reducing the risk of overfitting to a specific initialization.

#### Advantages of the proposed algorithm

The developed algorithm helps to increase the suggested model’s accuracy and decreases the computational complexity. Moreover, the optimal weighted feature selection using this algorithm helps to highlight the important features that are more informative regarding the presence of heart diseases. Further, the optimization of features and weights via the suggested EAVSRO algorithm helps to obtain accurate detection outcomes. The EAVSRO is not only used for optimal weighted feature selection but also for optimizing the parameters from AD-SVM and AMCCNet models. This process helps to improve the predictive power of suggested models for better diagnosis of cardiac disease. The pseudo-code of the developed EAVSRO is given in Algorithm 1.

**Algorithm 1:**
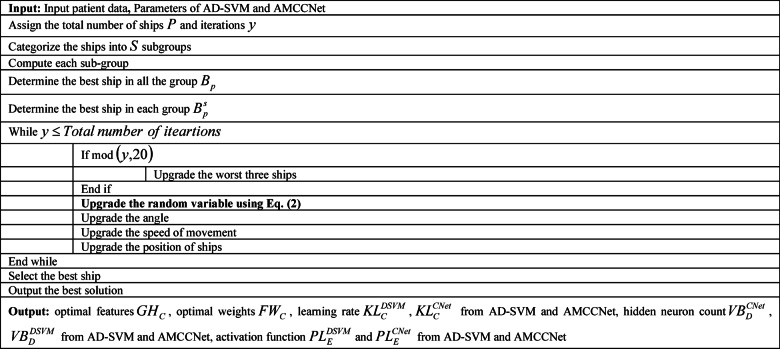
Suggested EAVSRO.

## Optimal feature selection and heart disease detection stage using deep learning

### Weighted optimal feature selection

The features from input data are retrieved by the EAVSRO algorithm to remove the irrelevant data to select only the useful features for heart disease detection. This dimensionality reduction results in a more compact and informative feature set, which simplifies the subsequent classification task and reduces the risk of overfitting. Furthermore, utilizing EAVSRO for assigning the optimized weights to the selected features eliminates unnecessary information from the input medical data. In addition to feature selection, EAVSRO allocates each feature with the best weight for reducing the impact of irrelevant features during classification. So, the suggested model concentrates more on crucial characteristics such as blood pressure, heart rate, and cholesterol levels for enhancing the accuracy of heart disease detection. By multiplying the optimal features by their corresponding optimal weights, the developed method can improve its ability to identify heart disease, even when the dataset is imbalanced.

For quicker and more accurate disease diagnosis, the weights are optimized and multiplied by the extracted features. The weighted optimal features$HW_{C}^{{OP}}$are computed using Eq. (3).

3 $$HW_{C}^{{OP}}=G{H_C}+F{W_C}$$

Here, the optimal features and optimal weights are indicated as$G{H_C}$and$F{W_C}$, respectively. The weights are optimized in the range of$\left[ {0.01 - 0.99} \right]$and the features lie in the interval of$\left[ {1 - 10} \right]$. The objective function${N_{j1}}$of the weighted optimal feature selection process is modeled below Eq. (4).

4 $${N_{j1}}=\mathop {\arg \hbox{min} }\limits_{{\left\{ {G{H_C},F{W_C}} \right\}}} \left( {\frac{1}{{{\chi ^2}}}} \right)$$

#### Chi-square

The maximized Chi-square represents the selected accurate features needed for heart disease detection. The normalized statistic between the observed and the expected feature selection outcome is determined by this measure. Based on the target, the features are selected by considering the chi-square scores. The formula for computing the chi-square statistic is given in Eq. (5).

5 $${\chi ^2}=\sum {\frac{{{{\left( {{A_x} - {G_x}} \right)}^2}}}{{{G_x}}}}$$

In the above expression, the expected and observed feature retrieval outcomes are denoted as ${G_x}$and${A_x}$, correspondingly.

### Deep SVM description

SVMs are robust computational models designed to handle both regression and classification challenges. The generalization capacity of the SVM^29^ model is high, as it is developed based on the principle of structural risk minimization. A kernel trick process is done by an SVM model that maps the original training data into a higher-dimensional feature space using kernel functions. Then, the SVM approach constructs an optimal hyperplane that separates data points from different classes. The hyperplane is defined as the margin between categories, and that is also known as support vectors. Non-linear and linear classification tasks are accomplished by the SVM model.

Consider the input attributes $\left\{ {{f_e}} \right\} \in {\Re ^\upsilon }$ and the output attributes $\left\{ {{g_e}} \right\} \in \Re$. So, for the dataset ${\left\{ {{f_e},{g_e}} \right\}_{e=1,....n}}$, the linear decision function is computed using Eq. (6).

6 $$d\left( f \right)=h,{\varphi _e}\left( f \right)+k$$

Here, the non-linear function is termed as $\varphi$and $k$ is a constant. Weight is represented as $h$. To minimize the function, two slack variables ${\ell _e}$and$\ell _{e}^{*}$are introduced as shown in Eq. (7).

7 $$L\left( {h,\ell _{e}^{*}} \right)=\frac{1}{2}{\left\| h \right\|^2}+P\sum\limits_{{e=1}}^{n} {\left( {{\ell _e}+\ell _{e}^{*}} \right)}$$

In Eq. (6), the error penalty parameter is given as $P$. In SVM, the Lagrangian multipliers are used for the final computation as given in Eq. (8).

8 $$d\left( f \right)=\sum\limits_{{e=1}}^{n} {\left( {{\mu _e} - \mu _{e}^{ * }} \right)} {M_{\ker }}\left( {{f_e},f} \right)+k$$

**Fig. 2:**
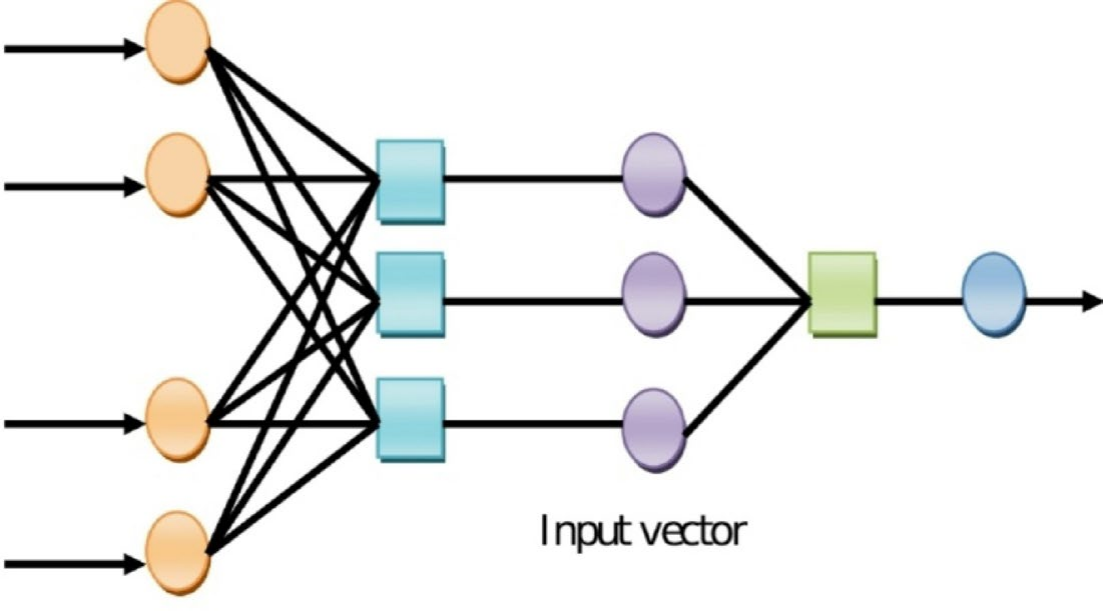
Structural representation of D-SVM.

Here, the RBF kernel is denoted as${M_{\ker }}\left( {{f_e},f} \right)$and the Lagrange multipliers are indicated as${\mu _e}$and$\mu _{e}^{ * }$. The prediction power of the SVM is dependent on the kernel parameter and the error penalty parameter. The structural representation of Deep SVM (D-SVM) is visualized in Fig. 2.

### Heart disease detection using AD-SVM

The proposed AD-SVM mechanism is capable of correctly categorizing intricate medical data for precise heart disease detection. The developed AD-SVM leverages the strength of traditional D-SVM.

#### Reasons for choosing the D-SVM

SVMs excel in dealing with datasets that have many features (high dimensionality), which is common in heart disease data, where factors like age, blood pressure, cholesterol levels, and other medical history contribute. Further, SVMs can use different kernel functions to map data into a higher-dimensional space, allowing them to model complex, non-linear relationships between features and the presence or absence of heart disease. However, SVMs can be computationally expensive with large datasets and may struggle with non-linear data. These limitations can be addressed by transforming them into D-SVMs. It utilizes deep learning techniques to improve performance, especially with complex, non-linear data patterns found in heart disease detection.

#### Novelty

The weighted optimal features$HW_{C}^{{OP}}$are given as input to the deep neural network. The deep network learns hierarchical features, progressively extracting more abstract and informative representations of the data at each layer. Due to the integration of deep learning layers in the SVM model, it can handle noisy and high-dimensional patient records by automatically extracting discriminative features from complex medical data. The extracted features from the deep network are then fed into the SVM classifier. D-SVM’s various hidden layers manage the feature correlation issues by capturing non-linear patterns in the weighted optimal features that improve the feature representation for robust heart disease detection outcomes. Furthermore, the optimization of hidden neuron count, learning rate and activation function using EAVSRO helps to overcome the variations or overlapping pattern issues in the patient data. The suggested AD-SVM is useful in discovering intricate dependencies and hidden patterns in the patient data. By modifying the learning parameters, the AD-SVM increases the model’s robustness and minimizes the overfitting problems. The AD-SVM effectively manages different factors, including blood pressure, cholesterol levels, and heart rate variations, for providing accurate heart disease detection. The D-SVM framework’s feature learning and decision boundary flexibility are improved with the use of EAVSRO for tuning the parameters. Thus, the probability of attaining misclassification outcomes is highly reduced. Finally, the developed AD-SVM offers the predicted heart disease outcome. Therefore, AD-SVM is benchmarked for early-stage cardiac disease diagnosis due to its deep-layered architecture along with precise parameter tuning. If the patient has heart disease, the target value 1 will be obtained as output, or else 0 will be obtained, indicating that the patient does not have cardiac disease. The objective function${N_{j2}}$of the EAVSRO-AD-SVM-aided heart disease detection model is given in Eq. (9).

9 $${N_{j2}}=\mathop {\arg \hbox{min} }\limits_{{\left\{ {G{H_C},F{W_C}} \right\}}} \left( {\frac{1}{{CSI}}+FPR} \right)$$

In the above equation, the optimized learning rate$KL_{C}^{{DSVM}}$ and hidden neuron count$VB_{D}^{{DSVM}}$ in DSVM are presented in between$\left[ {0.01 - 0.99} \right]$and$\left[ {5 - 255} \right]$. The activation function tuned in the range of $\left[ {1 - 5} \right]$is denoted by the term$PL_{E}^{{DSVM}}$. The Critical Success Index (CSI) and False Positive Rate (FPR) are determined using Eq. (10) and Eq. (11).

10 $$CSI=\frac{{{H_{ps}}}}{{\left( {{H_{ps}}+{G_{fs}}+{G_{fv}}} \right)}}$$

11 $$FPR=\frac{{{G_{fs}}}}{{{G_{fs}}+{H_{ns}}}}$$

Here, the false positive, true positive, true negative and false negative values are signified as${G_{fs}}$,${H_{ps}}$, ${H_{ns}}$and ${G_{fv}}$, respectively.

#### Discussion on how AD-SVM differs from traditional and kernel-based SVMs

**Fig. 3:**
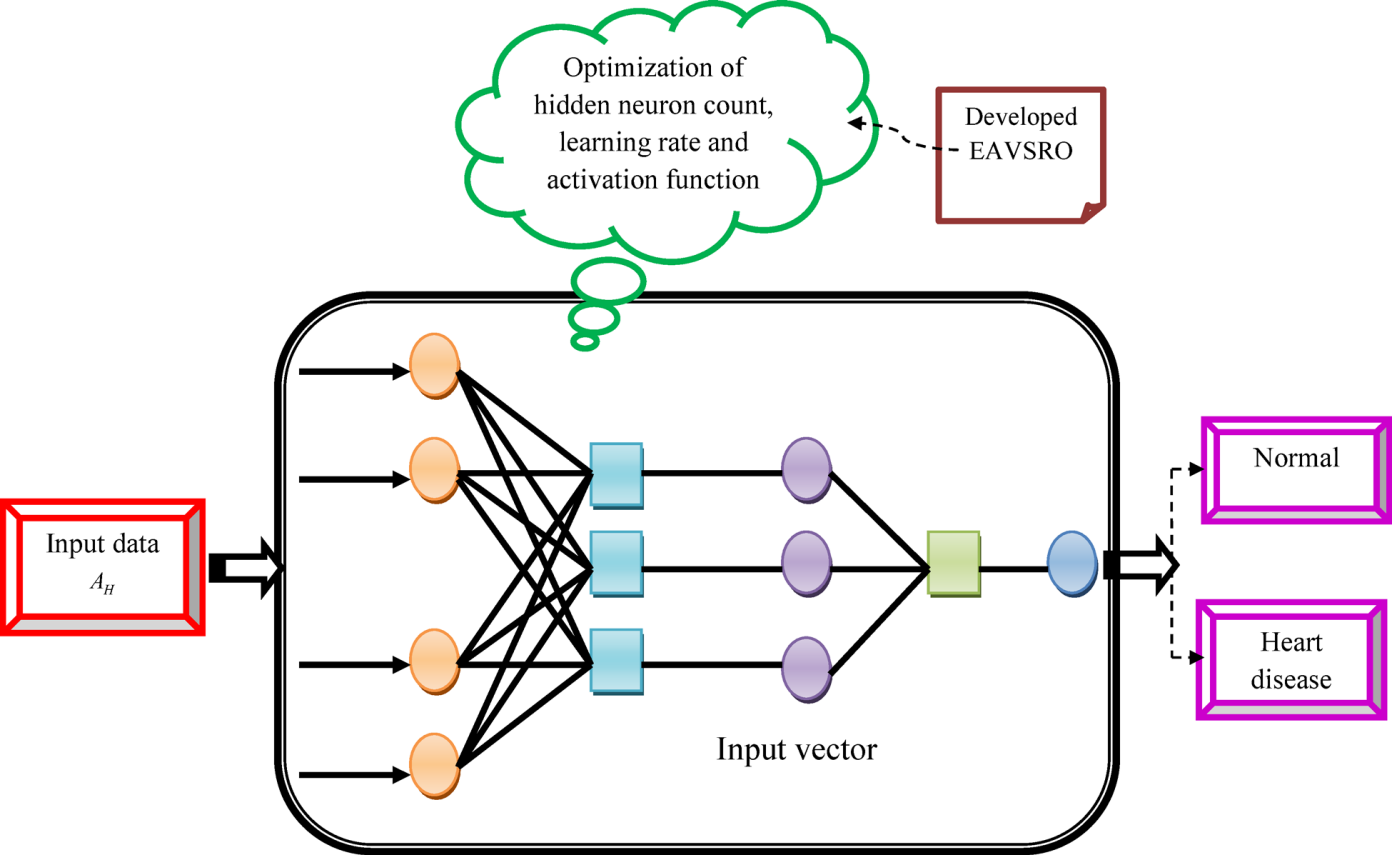
Structural framework of heart disease detection using AD-SVM.

The developed AD-SVM significantly differs from traditional^30^ or kernel-based SVMs^31^ in several key aspects. Firstly, traditional SVMs are shallow models that rely on hand-crafted features and kernel functions to map the data to a higher-dimensional space, whereas AD-SVM integrates deep learning techniques, such as neural networks, to automatically learn relevant features from the data. This allows AD-SVM to capture complex patterns and relationships in the data that may not be apparent to traditional SVMs. Additionally, AD-SVM employs an adaptive mechanism that adjusts the model’s parameters and architecture during training, based on the input data. This adaptability enables AD-SVM to handle varying levels of noise, outliers, and class imbalance in the data, which can be challenging for traditional SVMs. Moreover, AD-SVM can handle large datasets and high-dimensional feature spaces more efficiently than traditional SVMs. The unique combination of deep learning and adaptive mechanisms in the developed AD-SVM enables it to outperform the traditional SVM heart disease detection process. The structural framework of heart disease detection using AD-SVM is shown in Fig. 3.

## Multi-scale deep mechanism for atrial fibrillation rate determination from detected outcomes

### Description of CapsNet model

The architecture of CapsNet^32^ is categorized into two functional units: the encoder and the decoder. In this design, the encoder is responsible for feature extraction and is composed of three different layers. The fully connected layers are presented in the decoder, and they handle the input reconstruction. A spatial hierarchy-assisted dynamic routing technique is introduced by the CapsNet model for selectively sending a capsule’s outputs to appropriate capsules in the next layer by adjusting the routing weights.

#### Convolution layer

The low-level feature extraction process is handled by the convolution layer, and it is mathematically modeled in Eq. (12).

12 $${r_{ab}}=\sum\limits_{{v=1}}^{y} {\sum\limits_{{w=1}}^{z} {{g_{vw}}} } .{s_{a - v+1.b - w+1}}$$

In the above expression, the filter and the image matrix are denoted as${g_{vw}}$and${s_a}$, respectively.

#### Primary capsule layer

To process input features, the primary capsule layer converts the extracted features into vector form. The squashing activation function compresses the output vectors to reduce the computational requirements while enhancing the model’s ability to represent complex patterns. The mathematical derivation of the primary capsule layer is defined in Eq. (13).

13 $${v^{p\left( {a,b} \right)}}={g_f}\left( {\begin{array}{*{20}{c}} {{g_c}\left( {\sum\nolimits_{a} {c_{a}^{1}} } \right)} \\ \vdots \\ {{g_c}\left( {\sum\nolimits_{a} {c_{a}^{1}} } \right)} \end{array}} \right)$$

Here, the squash function is defined as${g_f}$. The convolution layer’s output is termed as${g_c}\left( {\sum\nolimits_{a} {c_{a}^{1}} } \right)$. The term${g_c}$indicates the function of the primary capsule layer, and the primary capsule is represented as${v^{p\left( {a,b} \right)}}$.

#### Digital capsule layer

Communication between the primary and digit capsules happens through a two-step mechanism. First, the vectors are linearly transformed to align with the target capsule dimensions. Then, the dynamic routing algorithm iteratively refines the connections to ensure that only the most relevant features are passed forward. When the high-level capsule $h$ receives data from the low-level capsule $m$, an activation function${\hat {v}_{b\left| a \right.}}$is attained as a result, and it is expressed in Eq. (14).

14 $${\hat {v}_{b\left| a \right.}}={z_{ab}}.{v_a}$$

The linear transformation matrix of the low-level capsules and output vectors are stated as${z_{ab}}$and${v_a}$, respectively. The squash function is represented in Eq. (15).

15 $${v_b}=\frac{{{{\left\| {{e_b}} \right\|}^2}}}{{1+{{\left\| {{e_b}} \right\|}^2}}}.\frac{{{e_b}}}{{\left\| {{e_b}} \right\|}}$$

**Fig. 4:**
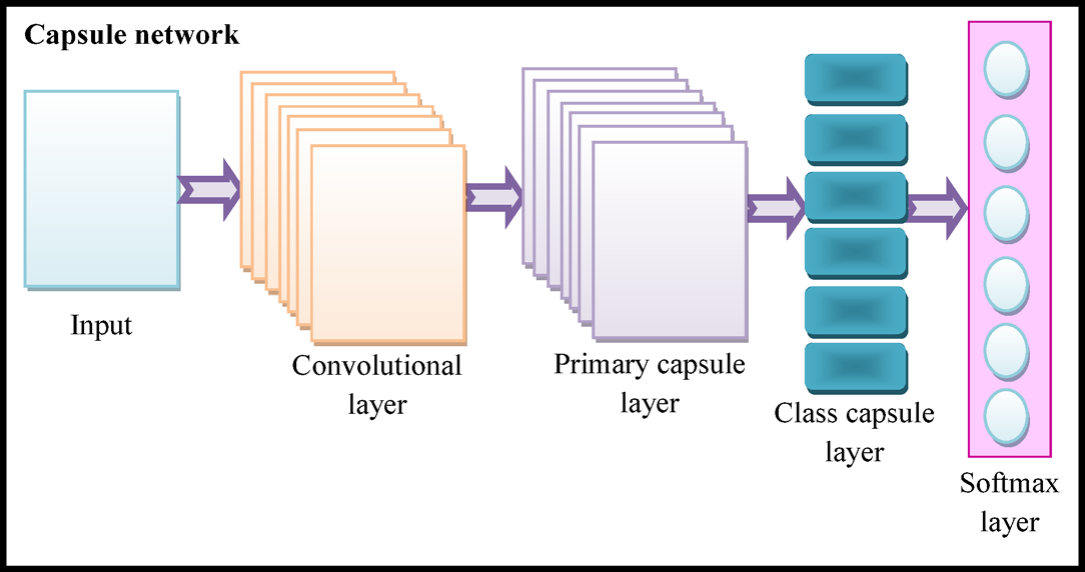
Pictorial depiction of CapsNet.

Here, the variable${e_b}$indicates the input after the weighted summation. A pictorial depiction of CapsNet is shown in Fig. 4.

### Atrial fibrillation rate determination using AMCCNet

The AF rate is identified using AMCCNet if the patient has heart disease.

#### Reasons for choosing CapsNet over conventional CNNs

The developed model selects the CapsNets due to its ability to excel at capturing and preserving spatial relationships between features, unlike CNNs like CNN–Resnet^33^, residual dense network with bidirectional recurrent neural network^34^, and CNN-ViT^35^ can lose this information through pooling layers. One of the primary limitations of traditional CNNs is their reliance on pooling operations, which discard valuable spatial information. This can lead to a loss of important details, particularly in medical imaging applications where subtle features can be critical for diagnosis. In contrast, CapsNet’s architecture is designed to preserve spatial hierarchies through its capsule structure. Each capsule represents a specific feature or object part, and the pose matrices capture the spatial relationships between these features. This allows CapsNet to maintain a richer representation of the data, enabling it to better capture complex relationships between features. In addition, CapsNet can handle small datasets, which is a common challenge in medical imaging applications. Traditional CNNs often require large amounts of training data to achieve optimal performance, but CapsNet’s architecture allows it to learn effective representations from smaller datasets. The unique strengths of CapsNet in handling spatial hierarchies and small datasets make it an attractive choice for medical imaging applications, such as detecting atrial fibrillation rates. By leveraging CapsNet’s strengths, the proposed framework can effectively capture complex patterns, ultimately leading to improved diagnosis and treatment of cardiac arrhythmias.

#### The rationale for combining multi-scale Convolution with CapsNet

In the conventional CapsNet architecture, the initial feature extraction stage relies on a single convolutional layer for capturing low-level details from the features. However, this simple approach leads to weak feature activation and unnecessary parameter overhead. To enhance the effectiveness of CapsNet, a multi-scale convolution is introduced in the proposed AMCCNet that increases the strength of the capsule generation process and improves the model’s ability to capture features at various scales. Replacing the traditional convolutional layer in CapsNet with a multi-scale convolutional structure enables the model to capture hidden features from the input. In the developed AMCCNet model, the integration of multi-scale convolution and CapsNet strengths is beneficial in enriching the feature diversity while minimizing the loss of essential information.

#### Working principle and novelty

Initially, the data from the diagnosed patient is fed into a convolutional layer, which extracts low-level features such as edges and textures. Specifically, the proposed framework employs convolutional kernels of varying sizes to capture the detailed structural features across multiple scales for determining the AF rate. This approach consists of convolution kernels with sizes$7 \times 7$, $5 \times 5$and $3 \times 3$for capturing both fine-grained and structural features. Furthermore, the network’s multiscale architecture enables it to capture features at different scales and resolutions, improving its representation learning capabilities. The outputs from two separate channels are combined to form a comprehensive feature. These features are then passed through primary capsule layers, which transform the features into capsules representing specific objects or patterns. In the primary capsule layer, the feature maps are processed and fused through convolution operations to form new capsule units. These newly formed capsules are then passed on to the digital capsule layer for attaining the AF rate prediction outcome. Furthermore, parameters like hidden neuron count, learning rate and the activation function of the AMCCNet method are optimized using the EAVSRO algorithm, enhancing the sensitivity of AF rate prediction by enabling dynamic feature extraction based on the complexity of input data.

#### Advantages of the AMCCNet

**Fig. 5:**
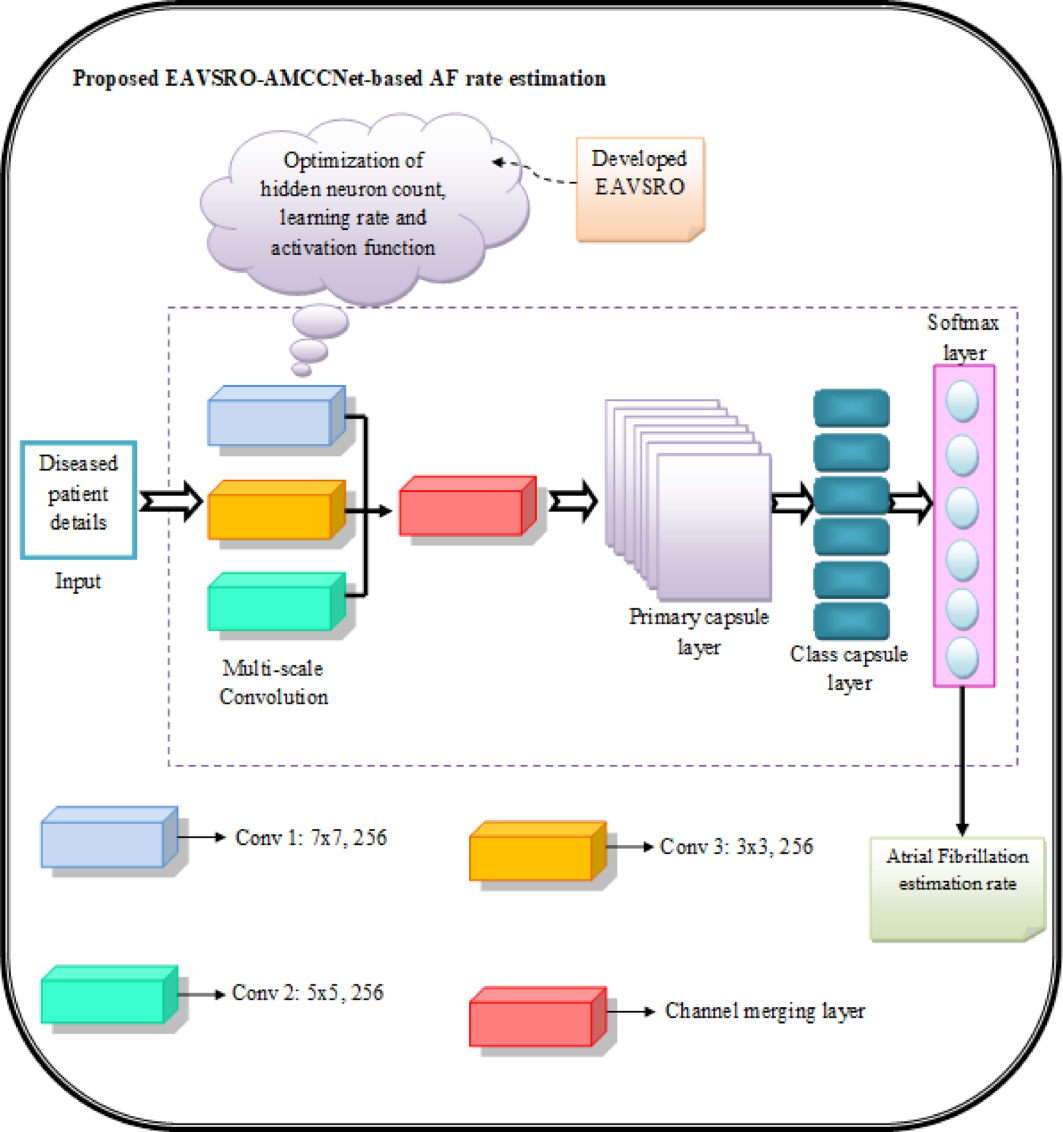
The structural layout of the proposed EAVSRO-AMCCNet-based AF rate estimation.

The proposed AMCCNet is suitable for learning intricate spatial and hierarchical patterns from medical data that are useful in obtaining precise AF rate prediction outcomes. Furthermore, the model’s multi-scale convolutional layers enable feature extraction at various resolutions for analyzing wide-level patterns. Both local and global patterns can be captured by the model because of the inclusion of multi-scale convolutional layers. Additionally, AMCCNet’s use of capsule layers provides a major benefit of capturing spatial hierarchies in the retrieved features. Thus, using the AMCCNet model for AF rate prediction helps with individualized patient monitoring and lowers the risk of serious cardiac events. The structural layout of the proposed EAVSRO-AMCCNet-based AF rate estimation is visualized in Fig. 5.

### Objective function

To increase the effectiveness and precision of AF rate estimation, parameter optimization in the AMCCNet model is conducted by using the EAVSRO algorithm. The complexity of medical data is handled by fine-tuning the parameters of the AMCCNet model. This optimization is useful for ensuring that the AMCCNet model learns efficient features from the input. Additionally, by balancing the model’s learning rate and activation function, the issues of overfitting and underfitting are reduced as this optimization procedure enhances the model’s capacity to generalize. Additionally, the optimization of parameters from the capsule layers can precisely maintain the spatial correlations between features, and the multi-scale convolution layers can efficiently extract the required information for AF rate estimation. This optimization procedure in AMCCNet during the AF rate estimation is helpful for decreasing the computational overhead issue and speeding up the detection process. The objective function${N_{j3}}$of the AMCCNet-assisted AF rate detection is given in Eq. (16).

16 $${N_{j3}}=\mathop {\arg \hbox{min} }\limits_{{\left\{ {KL_{C}^{{CNet}},VB_{D}^{{CNet}},PL_{E}^{{CNet}}} \right\}}} \left( {RMSE} \right)$$

In the above equation, the optimized learning rate$KL_{C}^{{CNet}}$ and hidden neuron count$VB_{D}^{{CNet}}$in AMCCNet are presented in between$\left[ {0.01 - 0.99} \right]$and$\left[ {5 - 255} \right]$. The activation function tuned in the range of $\left[ {1 - 5} \right]$is denoted by the term$PL_{E}^{{CNet}}$. RMSE is calculated via Eq. (17).

17 $$RMSE=\sqrt {\frac{{\sum\nolimits_{{b=1}}^{K} {{{\left( {{r_b} - {{\hat {r}}_b}} \right)}^2}} }}{K}}$$

In the above expression, the estimated and the actual scores are denoted as${\hat {r}_b}$ and${r_b}$, concurrently. The data points are symbolized as $K$.

## Evaluation results and discussion

### Experimental setup

The proposed heart disease detection with AF rate estimation model was implemented using Python. The initialization process was accomplished by considering a population range of 10, a chromosome length of 3 and a maximum iteration of 50. For the detection process, classifiers like CNN-LSTM^18^, DCNN^19^, Adaboost^20^, and SVM^29^ were considered. The algorithms used for the simulation process were Northern Goshawk Optimization (NGO)^36^, Remora Optimization Algorithm (ROA)^37^, African Bison Optimization Algorithm (ABOA)^38^ and SRO^28^. AF rate estimation performance was validated among models like GCN^21^, Recurrent Neural Networks (RNN)^39^, Gated Recurrent Units (GRU)^25^ and CapsNet^32^.

#### Reasons for choosing the existing models for comparison

In the designed heart disease detection with AF rate estimation model, conventional techniques such as CNN-LSTM, DCNN, CapsNet, and SVM are popular choices for comparison due to their ability to handle different data types and complexities. Specifically, CNN-LSTM is often a strong competitor in tasks like classification. CNN-LSTM combines the spatial feature extraction capabilities of CNNs with the sequential data processing power of LSTMs, making it suitable for tasks involving both spatial and temporal information. Further, CapsNets and DCNN can capture the spatial relationships between features in an image, particularly in recognizing objects. In addition, SVMs serve as a benchmark to compare the performance of more complex deep learning models, especially in tasks where simpler, handcrafted features can be effective. By contrasting the performance of CNN-LSTM, DCNN, CapsNet, and SVM against a developed approach, researchers can highlight the benefits of using deep learning for tasks like heart disease detection with AF rate estimation. Table 2 shows the hyperparameters of the existing model.

**Table 2:** Hyper-parameter settings for the existing model.

Model	Architecture Details / Base	Optimizer	Loss Function	Learning Rate	Activation	Batch size
CNN-LSTM^18^	CNN feature extractor + LSTM	Adam	Binary Cross-Entropy	0.001	ReLU (CNN), tanh (LSTM)	32
DCNN^19^	Deep CNN (4–5 conv + dense layers)	Adam	Cross-Entropy	0.0005	ReLU / Softmax	32
AdaBoost^20^	Ensemble of shallow Decision Trees	-	-	-	-	-
SVM^29^	RBF Kernel SVM	-	Hinge Loss	-	-	-
EAVSRO-AD-SVM	Enhanced Adaptive Voting + SVM	Adam	Binary Cross-Entropy	0.001	ReLU / Sigmoid	16

### Performance indicators

Various performance indicators, including precision, F1-score, MCC and accuracy, are utilized for evaluating the performance of heart disease detection. The AF rate estimation performance was validated by considering measures like Mean Percentage Error (MPE), Mean Absolute Error (MAE) and Symmetric Mean Absolute Percentage Error (SMAPE). The calculations for all the metrics are given in Eq. (17)-Eq. (23).

18 $$Accuracy=\frac{{{H_{ps}}+{H_{ns}}}}{{{H_{ps}}+{H_{ns}}+{G_{fs}}+{G_{fv}}}}$$

19 $$\Pr ecision=\frac{{{H_{ps}}}}{{{H_{ps}}+{G_{fs}}}}$$

20 $$F1score=\frac{{2{H_{ps}}}}{{2{H_{ps}}+{G_{fs}}+{G_{fv}}}}$$

21 $$MCC=\frac{{{H_{ps}} \times {H_{ns}} - {G_{fs}} \times {G_{fv}}}}{{\sqrt {\left( {{H_{ps}}+{G_{fs}}} \right)\left( {{H_{ps}}+{G_{fv}}} \right)\left( {{H_{ns}}+{G_{fs}}} \right)\left( {{H_{ns}}+{G_{fv}}} \right)} }}$$

22 $$SMAPE=\frac{1}{k}\sum\limits_{1}^{k} {\frac{{\left| {\hat {r} - r} \right|}}{{\frac{{\left( {\left| {\hat {r}} \right|+\left| r \right|} \right)}}{2}}}}$$

23 $$MAE=\frac{1}{k}\sum\limits_{{b=1}}^{k} {\left| {r - \hat {r}} \right|}$$

24 $$MPE=\frac{{\sum {\frac{{\left| {\hat {r} - r} \right|}}{r}} }}{K}$$

### Accuracy estimation of heart disease detection

**Fig. 6:**
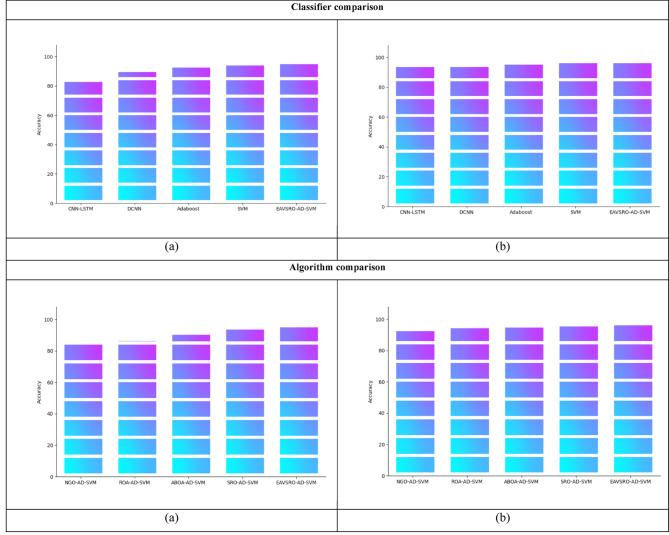
Accuracy Assessment of the Proposed Heart Disease Detection Model concerning (**a**) Dataset 1 and (**b**) Dataset 2.

The AD-SVM model is compared with CNN-LSTM, DCNN, Adaboost and standard SVM models by utilizing a k-fold analysis to validate its performance in heart disease detection. The suggested AD-SVM framework outperforms the current models as shown by the graphical results in Fig. 6. The precision of CNN-LSTM, DCNN and Adaboost models is lower than the proposed model as they have a limited ability to handle high-dimensional medical data. Moreover, the existing techniques struggle to identify the intricate patterns present in datasets, which leads to poor accuracy as shown in the graphs. The accuracy of EAVSRO-AD-SVM in dataset 1 is 12.7%, 7.44%, 5.31% and 2.12% higher than CNN-LSTM, DCNN, Adaboost and SVM. Here, the EAVSRO optimally selects the discriminative features while removing unnecessary data that is highly useful for the improvement of AD-SVM’s performance.

### Numerical evaluation of heart disease detection

The suggested AD-SVM model for heart disease prediction is evaluated with other traditional models and algorithms, as shown in Tables 3 and 4. The accuracy and precision are compared across several learning optimizers like Adam, SGD, RMSprop, Adagrad, and gradient descent. Traditional classifiers such as CNN-LSTM and DCNN attained less accuracy because of their poor feature learning capabilities and their inability to manage the intricate nonlinear patterns in heart disease datasets. Moreover, other techniques like Adaboost also sufficiently attained better precision scores in heart disease detection. The suggested AD-SVM model can identify important patterns and minute variations in patient data. The accuracy of AD-SVM-aided heart disease detection at Adam and RMSprop optimizers is 96.58% and 94.92% for dataset 2. The model reaches its maximum accuracy with optimizers like Adagrad and Adam, which shows its high learning efficiency in different weighted features. Thus, the AD-SVM model’s high prediction accuracy over conventional models shows its usefulness in cardiac disease detection applications for preventing issues like heart failure.

**Table 3:** Numerical evaluation of heart disease detection performance using dataset 1.

Optimizers	NGO-AMCCNet^36^	ROA-AMCCNet^37^	ABOA-AMCCNet^38^	SRO-AMCCNet^28^	EAVSRO-AD-SVM
**Dataset 1**					
**Algorithm comparison**					
**Accuracy**					
Adam	85.95579	87.25618	89.85696	92.58778	94.92848
SGD	84.53453	88.13814	90.84084	92.34234	94.29429
RMSprop	84.75177	89.00709	91.13475	93.26241	94.32624
Adagrad	85.15625	85.9375	89.84375	92.57813	95.70313
Gradient Descent	86.07242	88.30084	89.41504	91.08635	92.20056
**Precision**					
Adam	84.44976	86.16505	88.4058	91.85185	93.64303
SGD	82.22222	85.55556	88.48315	90.8046	92.55014
RMSprop	82.45033	87.16216	89.72603	92.06897	93.10345
Adagrad	83.46457	83.20611	88.18898	90.625	95.16129
Gradient Descent	83.51064	84.53608	88.33333	91.37931	90.65934
**MCC**					
Adam	87.74929	88.51541	91.5493	93.40659	96.38889
SGD	87.2549	91.17647	93.54839	94.02516	96.21451
RMSprop	87.40458	91.04478	92.64706	94.52555	95.62044
Adagrad	86.82171	88.8	91.47287	94.53125	96.21212
Gradient Descent	88.88889	92.72727	90.50279	90.81081	93.78531

Optimizers	CNN-LSTM^18^	DCNN^19^	Adaboost^20^	SRO-AMCCNet^28^	EAVSRO-AD-SVM
**Classifier comparison**					
**Accuracy**					
Adam	85.43563	89.3368	91.6775	94.01821	94.92848
SGD	87.08709	89.03904	93.09309	94.29429	94.29429
RMSprop	87.41135	89.3617	90.60284	94.32624	94.32624
Adagrad	84.375	89.84375	90.23438	92.1875	95.70313
Gradient Descent	84.95822	88.85794	92.47911	94.70752	92.20056
**Precision**					
Adam	83.97129	88.1068	90.09662	93.75	93.64303
SGD	85.26912	87.86127	91.64265	93.04348	92.55014
RMSprop	86.00683	87	88.55219	93.10345	93.10345
Adagrad	82.17054	87.5969	86.56716	89.31298	95.16129
Gradient Descent	81.77083	86.95652	90.27027	94.85714	90.65934
**MCC**					
Adam	87.17949	90.7563	93.52113	94.30894	96.38889
SGD	89.13738	90.3125	94.67085	95.63863	96.21451
RMSprop	88.92989	92.04545	92.8839	95.62044	95.62044
Adagrad	86.61417	92.12598	94.2623	95.2	96.21212
Gradient Descent	87.17949	90.7563	93.52113	94.30894	96.38889

**Table 4:** Numerical evaluation of heart disease detection performance using dataset 2.

Optimizers	NGO-AMCCNet^36^	ROA-AMCCNet^37^	ABOA-AMCCNet^38^	SRO-AMCCNet^28^	EAVSRO-AD-SVM
**Dataset 2**					
**Algorithm comparison**					
**Accuracy**					
Adam	92.72464	93.33333	94.66667	95.53623	96.37681
SGD	92.87625	93.84615	94.24749	95.38462	96.58863
RMSprop	92.6087	93.28063	95.05929	95.41502	96.20553
Adagrad	92.6087	93.21739	94.95652	94.95652	96.95652
Gradient Descent	92.1118	94.09938	94.65839	95.15528	96.70807
**Precision**					
Adam	79.07285	80.66667	84.65753	87.43017	88.75171
SGD	78.86057	82.75316	83.70253	86.6242	89.87138
RMSprop	78.03163	80.88235	85.60748	87.21374	89.88327
Adagrad	79.83539	79.6875	85.83333	85.53719	91.48936
Gradient Descent	78.76106	81.97183	82.96089	86.09467	91.38462
**MCC**					
Adam	96.54917	96.85185	97.35294	97.65911	98.4197
SGD	96.90056	96.81934	97.07379	97.7138	98.35304
RMSprop	96.83835	96.67674	97.59398	97.55733	97.81746
Adagrad	96.03087	97.09172	97.36264	97.46696	98.36066
Gradient Descent	95.6727	97.52988	98.00319	97.56289	98.05447

Optimizers	CNN-LSTM^18^	DCNN^19^	Adaboost^20^	SVM^29^	EAVSRO-AD-SVM
**Classifier comparison**					
**Accuracy**					
Adam	93.50725	93.7971	95.18841	96.02899	96.37681
SGD	93.11037	94.08027	94.94983	95.78595	96.58863
RMSprop	93.04348	93.99209	95.13834	95.92885	96.20553
Adagrad	93.47826	95.47826	94.17391	96.08696	96.95652
Gradient Descent	93.54037	93.10559	95.2795	95.90062	96.70807
**Precision**					
Adam	81.74387	82.51366	85.30997	88.34951	88.75171
SGD	80.06135	82.43451	85.41997	87.34177	89.87138
RMSprop	80.78358	82.89963	86.47619	88.52772	89.88327
Adagrad	81.1245	86.17886	84.68085	89.02954	91.48936
Gradient Descent	81.87135	80.40346	85.75581	87.64706	91.38462
**MCC**					
Adam	96.6863	96.83591	97.89513	98.0579	98.4197
SGD	96.74936	97.30884	97.49894	98.04919	98.35304
RMSprop	96.33902	96.98795	97.40648	97.8575	97.81746
Adagrad	96.89234	98.00885	96.61202	97.91895	98.36066
Gradient Descent	96.6877	96.59541	97.8673	98.11024	98.05447

**Fig. 7:**
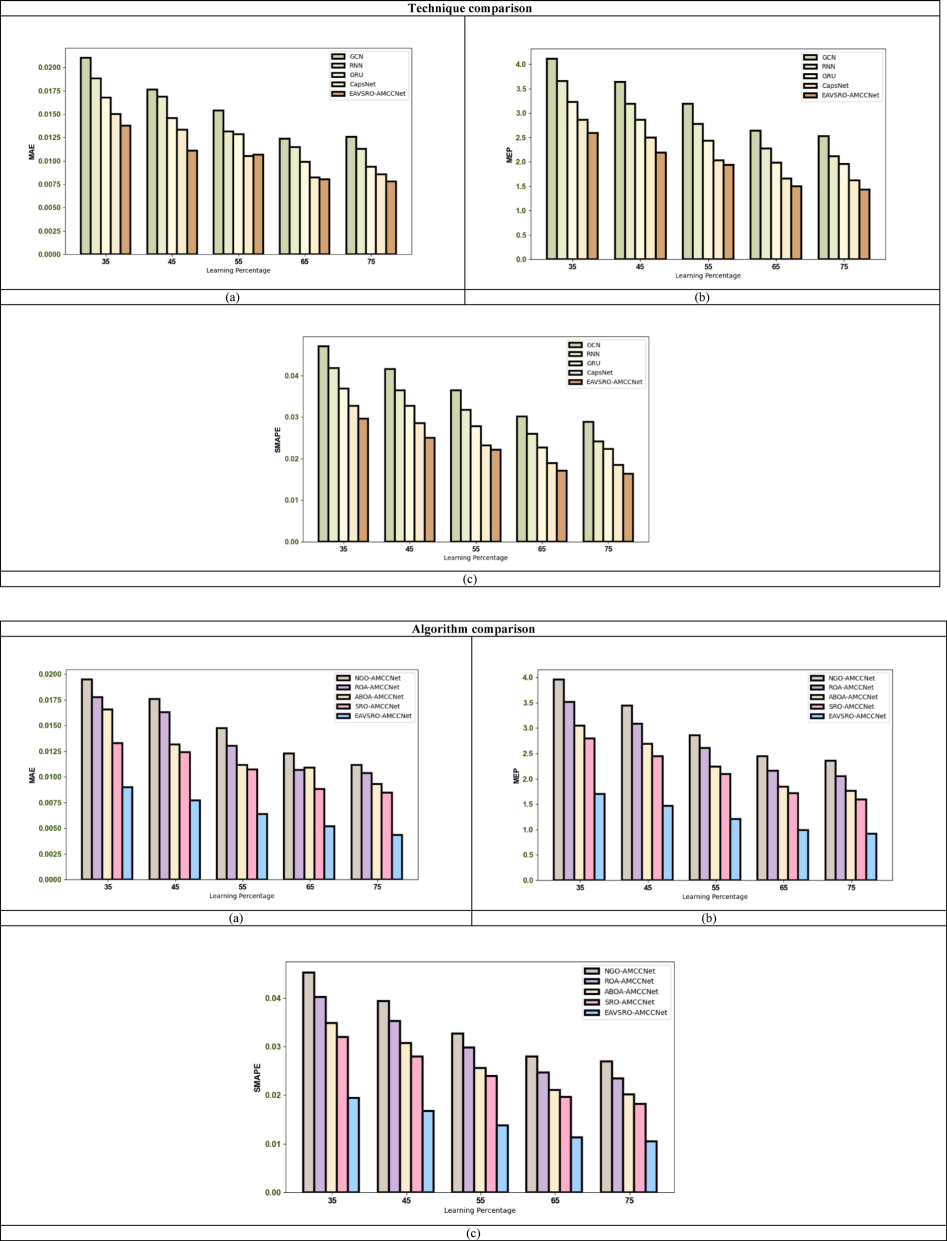
AF Rate Estimation Performance Validation on Dataset 1 in Terms of **a**) MAE, **b**) MEP and **c**) SMAPE.

### Atrial fibrillation prediction analysis

**Fig. 8:**
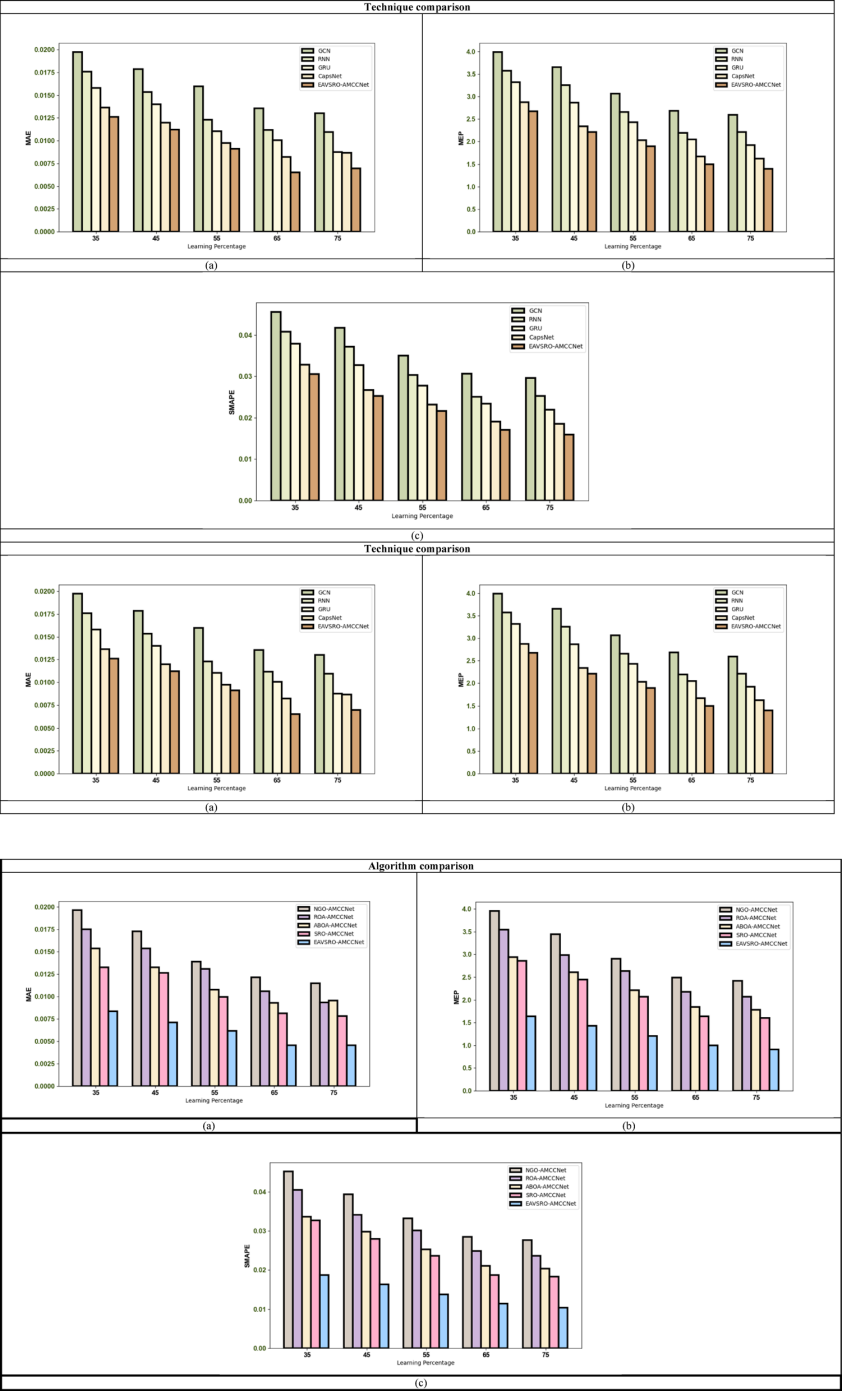
AF Rate Estimation Performance Validation on Dataset 2 in terms of **a**) MAE, **b**) MEP and **c**) SMAPE.

The AF prediction performance of the implemented AMCCNet is contrasted with other conventional models, and the results for dataset 1 and dataset 2 are visualized in Figs. 7 and 8. At a learning percentage of 55, the SMAPE of the recommended AMCCNet-based AF rate prediction is 8.33%, 25%, 30% and 58.33% better than GCN, RNN, GRU and CapsNet. These results confirm that the classical models like GCN and GRU have limited feature extraction capabilities, and they cannot retrieve temporal as well as spatial features without increasing the computational complexity. Even though RNN has high potential in handling sequential learning, it does not have a greater ability to handle non-linear data patterns. However, the proposed AMCCNet efficiently analyze the deep temporal patterns needed for the precise AF rate determination.

### Convergence analysis

**Fig. 9:**
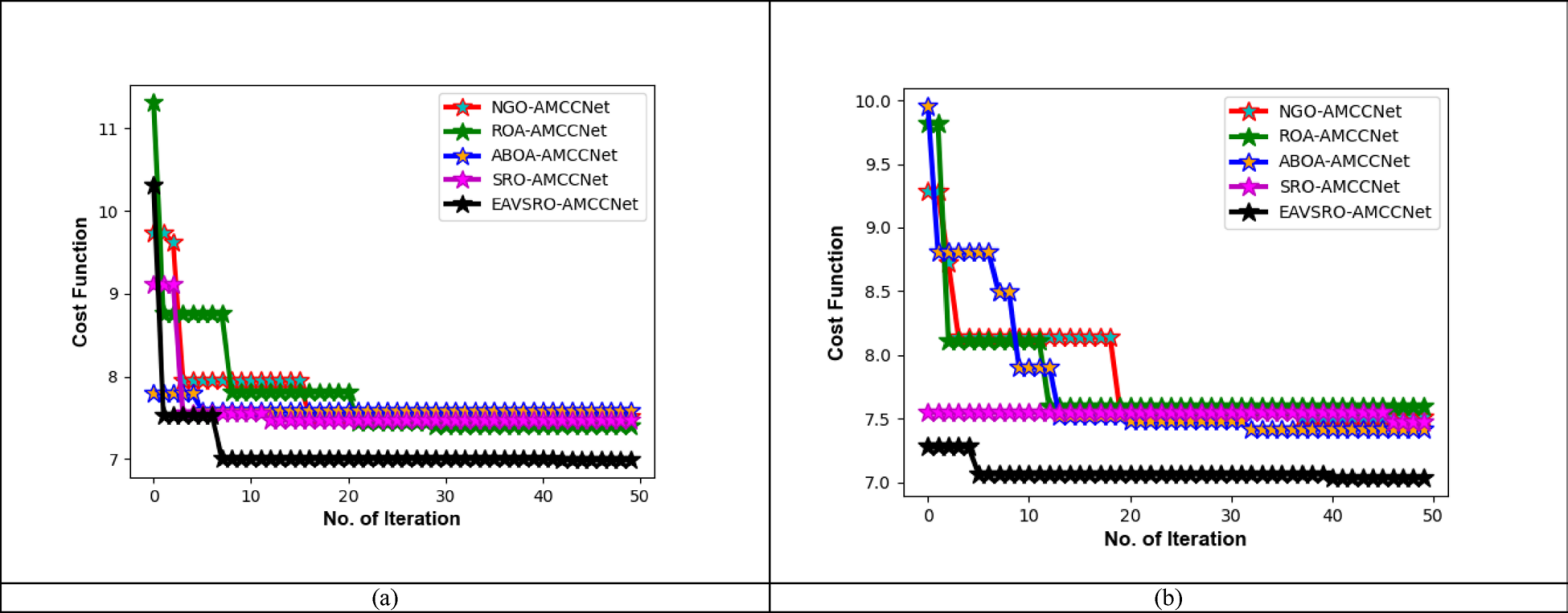
Convergence analysis of the developed heart disease detection model by means of a) Dataset 1 and Dataset 2.

The graphical outcomes in Fig. 9 show the suggested optimization technique’s performance in terms of convergence. The suggested method converges to a better solution in a few numbers of iterations, while algorithms like NGO-AMCCNet, ROA-AMCCNet, ABOA-AMCCNet and SRO-AMCCNet need more iterations to stabilize and increase the time taken to find an optimal solution. The improved exploration and exploitation capabilities of the suggested algorithm, due to the modification in random attribute calculation, are helpful for this better convergence. The suggested approach avoids premature convergence and escapes from local optima due to the random variable update concept based on different fitness measures. Both the global and local searching phases are enhanced in the proposed EAVSRO as per the results below. Thus, utilizing EAVSRO for parameter optimization is beneficial in enhancing the resilience and reliability of the prediction process.

### Statistical results

The suggested algorithm’s efficiency over traditional methods such as NGO-AMCCNet, ROA-AMCCNet, ABOA-AMCCNet and SRO-AMCCNet is seen from the statistical analysis provided for dataset 1 and dataset 2 in Table 5. Among all algorithms, the suggested model obtained better mean and standard deviation outcomes that demonstrate its capabilities in identifying the optimal solution throughout the search process. Existing algorithms like NGO and ROA have drawbacks like poor exploration-exploitation balance, or those heuristic strategies have the tendency to become caught in local optima. The suggested EAVSRO has effectively rectified these difficulties due to its new mechanism for computing random numbers. This process is useful in dynamic search, and it is confirmed by its lowest mean values. Furthermore, the suggested method’s reduced standard deviation number when compared to other approaches like ROA and NGO proves its consistency in optimal solution searching. This result shows the stability of EAVSRO in all iterations. Therefore, the statistical results show the efficiency of EAVSRO in performing precise weighted optimal feature selection.

**Table 5:** Statistical results of the implemented heart disease detection model among conventional Algorithms.

Measures	NGO-AMCCNet^36^	ROA-AMCCNet^37^	ABOA-AMCCNet^38^	SRO-AMCCNet^28^	EAVSRO-AMCCNet
**Dataset 1**					
BEST	7.517725	7.396697	7.580928	7.473193	6.994479
WORST	9.729048	11.31079	7.790808	9.104495	10.31477
MEAN	7.75988	7.78004	7.601916	7.585561	7.135434
MEDIAN	7.517725	7.443064	7.580928	7.473193	7.009552
STANDARD DEVIATION	0.52302	0.681126	0.062964	0.384979	0.484674
**Dataset 2**					
BEST	7.508781	7.592237	7.41951	7.468669	7.036341
WORST	9.28713	9.82213	9.951201	7.549708	7.278949
STANDARD DEVIATION	0.430635	0.464763	0.56486	0.021985	0.066813
MEDIAN	7.559547	7.592237	7.478387	7.549708	7.066501
MEAN	7.825672	7.785971	7.746062	7.543225	7.081714

### Comparison of developed model with traditional machine learning classifiers and recent deep learning models

Table 6 shows the comparison of the developed model with traditional machine learning classifiers and recent deep learning models. This analysis helps to reveal the strengths and weaknesses of each method, leading to the development of more robust and reliable AF detection systems. The accuracy of the developed EAVSRO-AD-SVM is 7.86%, 5.73%, and 8.77% enhanced than the other deep learning techniques, such as GCN, RawECGNet, and O-SBGC-LSTM, respectively. Thus, the results proved that the parameter optimization procedures in the developed AD-SVM enhance the model’s ability to handle the non-linear patterns in the input datasets, demonstrating its superior capability in heart disease prediction.

**Table 6:** Comparison of developed model with traditional machine learning classifiers and recent deep learning models.

Deep learning technique				
Methods	GCN^21^	RawECGNet^22^	O-SBGC-LSTM^3^	EAVSRO-AD-SVM
**Accuracy**				
Adam	89.35	91.15	88.6	96.37681
SGD	87.6	90.425	87.975	96.58863
RMSprop	88.875	89.975	87.425	96.20553
Adagrad	89.675	91.45	90.625	96.95652
Gradient Descent	88.725	91.475	90.525	96.70807
**Precision**				
Adam	89.3	91.3	88.8	88.75171
SGD	87.3	90.3	87.9	89.87138
RMSprop	89.4	89.9	87.4	89.88327
Adagrad	89.8	91.6	90.7	91.48936
Gradient Descent	89.1	91.7	90.5	91.38462
**MCC**				
Adam	0.741	0.782	0.725	98.4197
SGD	0.702	0.765	0.711	98.35304
RMSprop	0.732	0.755	0.699	97.81746
Adagrad	0.749	0.789	0.770	98.36066
Gradient Descent	0.729	0.790	0.768	98.05447

Machine learning technique				
Optimizers	TML-EML^13^	Machine learning- based framework^26^	LS-SVM^27^	SVM^29^
**Accuracy**				
Adam	87.600	91.275	89.400	96.37681
SGD	89.425	89.775	89.150	96.58863
RMSprop	88.150	88.650	88.575	96.20553
Adagrad	89.425	91.200	90.975	96.95652
Gradient Descent	88.525	91.850	90.475	96.70807
**Precision**				
Adam	87.400	91.200	89.200	88.75171
SGD	89.800	89.600	89.100	89.87138
RMSprop	88.100	88.300	88.700	89.88327
Adagrad	89.500	91.200	91.000	91.48936
Gradient Descent	88.600	92.100	90.400	91.38462
**MCC**				
Adam	0.702	0.785	0.742	98.4197
SGD	0.744	0.750	0.737	98.35304
RMSprop	0.715	0.725	0.725	97.81746
Adagrad	0.743	0.783	0.778	98.36066
Gradient Descent	0.723	0.799	0.766	98.05447

### Generalizability analysis

**Fig. 10:**
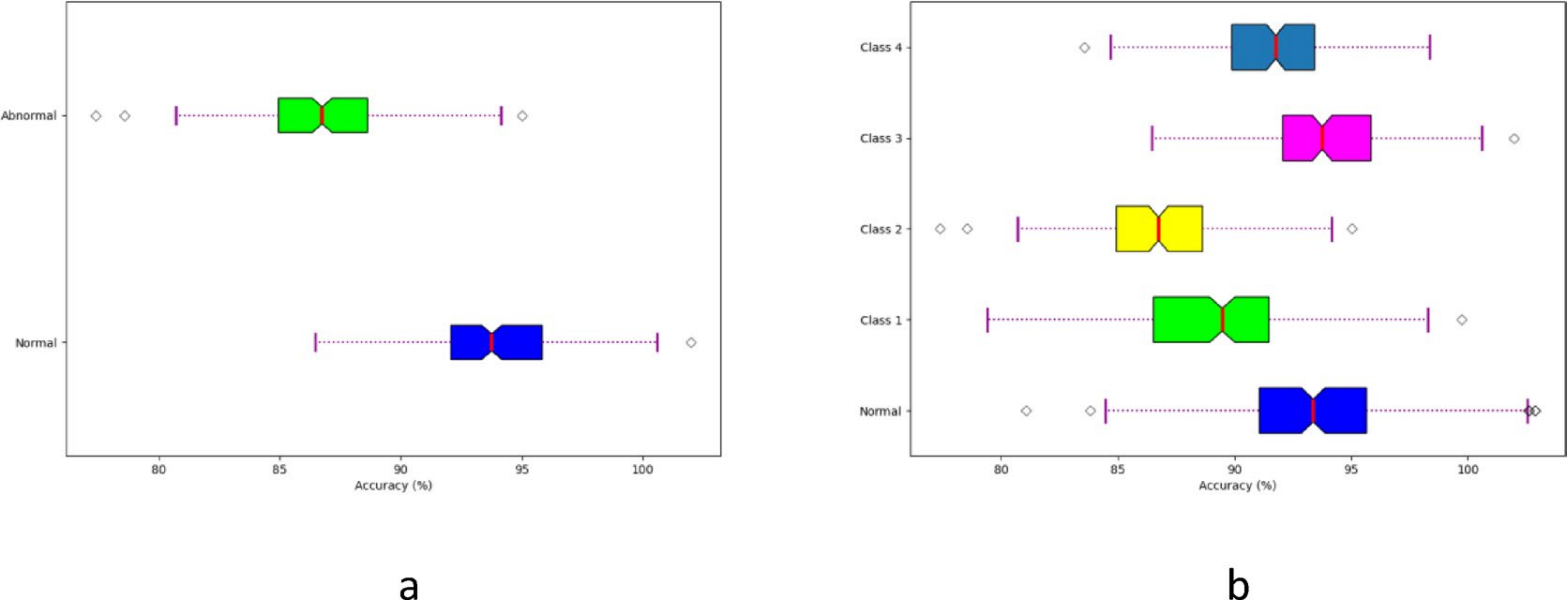
Generalizability analysis of the developed heart disease detection model in terms of a) Dataset 1 and Dataset 2.

The generalizability analysis of the developed model is shown in Fig. 10. The analysis across different datasets helps assess how well the model performs on unseen data, ensuring its reliability and clinical utility. This analysis helps determine if the model’s performance is consistent across various patient populations, recording conditions, and data sources, which is crucial for its adoption in diverse clinical settings. The parameter optimization process employed in the developed model helps to learn from various datasets and identify the most relevant patterns and features. This adaptability enables the model to generalize well across different datasets, even those with varying characteristics and distributions.

### Complexity analysis

The complexity analysis of the developed model is shown in Table 7. The EAVSRO’s feature weighting mechanism enables the framework to assign optimal weights to the selected features, further enhancing the accuracy and efficiency of the classification task. The complexity of the developed model is${\text{O[MI + nn + Ch]}}$. Thus, the outcome proved that by selecting the most relevant features and tuning their weights, EAVSRO reduces the complexity of the data, making it easier to classify and detect heart diseases. This efficient feature selection and weighting technique enables the framework to achieve improved performance while reducing computational complexity, ultimately leading to a more streamlined and effective approach to heart disease detection.

**Table 7:** Complexity analysis.

Algorithm	Complexity
NGO-AMCCNet^36^	${\text{O[MI + 2 + nn + 3 + Ch + 2]}}$Here, the term ${\text{MI }}$represents the maximum iteration, ${\text{nn }}$signifies the number of population, and ${\text{Ch}}$depicts the chromosome length
ROA-AMCCNet^37^	${\text{O[MI + 3 + nn + 1 + Ch + 1]}}$
ABOA-AMCCNet^38^	${\text{O[MI + 2 + nn + 2 + Ch + 2]}}$
SRO-AMCCNet^28^	${\text{O[MI + 1 + nn + 1 + Ch]}}$
EAVSRO-AMCCNet	${\text{O[MI + nn + Ch]}}$

### Clinical deployment of the developed model

The proposed heart disease detection model has significant potential for clinical deployment and integration into real-world clinical settings. Firstly, interpretability is crucial, as clinicians need to trust and understand the model’s decisions and predictions. To achieve this, the developed model employs AMCCNet techniques. This approach enhances interpretability by combining the feature extraction capabilities of convolutional layers with the part-based representation power of capsules, enabling a more nuanced understanding of spatial hierarchies and relationships within data. This transparency will enable clinicians to confidently rely on the model’s output, making informed decisions that ultimately improve patient outcomes. Furthermore, the developed model can effectively integrate into Clinical Decision-Support Systems (CDSSs) to assist healthcare professionals in making more accurate and timely diagnoses and treatment plans. The integration of D-SVM in the developed AD-SVM models can effectively analyze patient data to predict the likelihood of heart disease, identify high-risk individuals, and suggest potential interventions. This integration can enhance diagnostic accuracy and improve overall patient care. By prioritizing interpretability and integration, the proposed heart disease detection model can be successfully deployed in clinical settings, providing valuable support to clinicians and improving patient outcomes.

## Conclusion

A heart disease prediction framework has been developed in this research by integrating machine learning and deep learning models. The required clinical data were collected from standard benchmark datasets. The EAVSRO algorithm was utilized to extract relevant features and eliminate noisy or irrelevant features. Additionally, EAVSRO was employed to assign optimal weights to the selected features to prevent the feature dimensionality issues and improve computational efficiency. The weighted optimal features were then subjected to the AD-SVM model for accurate heart disease classification. For patients with heart disease, the AF rate was determined using the implemented AMCCNet model. The AMCCNet effectively extracted multi-level and hierarchical features to predict the AF rate. Furthermore, the parameters of both AD-SVM and AMCCNet models were fine-tuned using the EAVSRO algorithm to enhance the overall prediction performance. Finally, the proposed framework was validated, and its performance was compared with conventional models using various evaluation metrics. The accuracy of the suggested model was 96.7% for heart disease detection while analyzing with a gradient descent optimizer. Thus, the proposed mechanism is beneficial in monitoring cardiac health conditions efficiently. Despite its promising results, the proposed model is required to acknowledge that real-world validation and model interpretability in clinical settings remain as future work. The proposed framework’s performance was also not validated using real-world clinical data, involving diverse patient populations and varying data quality. Moreover, the interpretability of the model’s predictions is crucial in clinical settings, where understanding the underlying reasons for a diagnosis is essential for decision-making. Future studies will focus on addressing these limitations, exploring techniques to improve model interpretability, such as feature attribution and explanation methods, and conducting extensive clinical validation to ensure the framework’s efficacy in real-world applications. Moreover, in future work, other cardiovascular conditions like hypertension, stroke risk, or arrhythmia detection will be performed with advanced deep learning approaches, and future extensions of the developed model will focus on fusing data from mobile^4^ and auscultation-based methods^25^, paving the way for more accurate, efficient, and personalized heart disease detection and management. Furthermore, privacy-preserving techniques will be explored to maintain the confidentiality of patient data, and preprocessing will be performed on the developed model to clean and prepare the data for analysis, improve model performance, and ensure accurate predictions.

### Deployment of proposed model in real-time systems or mobile devices

To expand the model’s potential for real-time applications, the designed approach can be integrated into the mobile health monitoring systems or wearable systems to enable early disease detection and continuous cardiac health assessment. By optimizing the model for lightweight deployment, it could execute effectively on embedded systems or mobile processors. This would allow for the real-time processing of data garnered from the sensors, such as smart watches or ECG monitors, providing user’s immediate feedback on their cardiac status and alerting them or healthcare providers in case of abnormalities. Moreover, combining the model into telemedicine platforms could improve personalized care and remote diagnostics, most importantly in rural or resource-limited areas. Such deployments would require guaranteeing energy efficiency, low latency, and secure data handling to maintain the system reliability and user privacy.

## Data Availability

Two different datasets are considered in this research for the diagnosis of heart disease.Dataset 1 (Heart Disease Dataset): This dataset is accessed on 2025-04-08 from https://www.kaggle.com/datasets/johnsmith88/heart-disease-dataset. This database consists of different attributes that specify the presence of heart disease. Among 76 attributes, 14 subsets of detail including blood pressure, sugar, cholesterol and other attributes are presented in this dataset. Dataset 2 (heart disease): This multivariate database is available on https://archive.ics.uci.edu/dataset/45/heart+disease and it is accessed on 2025-04-08. This dataset possesses numerous details including the patient’s smoking history, chest pain details and so on.
